# 

**Published:** 1982

**Authors:** 


### Contents

4 **Images and Measurements in Clinical Radiology**
E. Rhys Davies

12 **The Natural History of Cowpox**
Derrick Baxby

17 **Corneal Transplantation in Bristol, 1970-80**
Andrew Tullo

21 **Obituary**
Mr. Douglas Mearns Milne

22 **Abstracts of Meeting**
Autumn Meeting of the Surgical Club of South West England, held at Gloucester and Cheltenham on 6th and 7th November 1981

28 **Book Review**
Essential Medicine – J. L. Burton

29 **Examination Results**

